# Does the protocol-required uniform margin around the CTV adequately account for setup inaccuracies in whole breast irradiation?

**DOI:** 10.1186/s13014-021-01863-w

**Published:** 2021-08-03

**Authors:** Jurui Luo, Zhihai Yin, Zhen Zhang, Xiaomao Guo, Xiaoli Yu, Juanqi Wang

**Affiliations:** 1 Department of Radiation Oncology, Fudan University Shanghai Cancer Center, 270 DongAn Road, Shanghai, China; 2 Department of Oncology, Shanghai Medical College, Fudan University, Shanghai, 200032 China

**Keywords:** Whole breast irradiation, Cone-beam computed tomography, Setup error, Patient characteristics, Margin selection

## Abstract

**Purpose:**

To use cone-beam computed tomography (CBCT) imaging to determine the impacts of patient characteristics on the magnitude of geometric setup errors and obtain patient-specific planning target volume (PTV) margins from the correlated patient characteristics in whole breast irradiation (WBI).

**Methods:**

Between January 2019 and December 2019, a total of 97 patients who underwent breast-conserving surgery, followed by intensity-modulated radiation therapy in WBI, were scanned with pre-treatment CBCT for the first three treatment fractions and weekly for the subsequent fractions. Setup errors in the left–right (LR), superior–inferior (SI) and anterior–posterior (AP) directions were recorded and analyzed with patient characteristics—including age, tumor location, body mass index (BMI), chest circumference (CC) and breast volume (BV)—to examine the predictors for setup errors and obtain specific PTV margins.

**Results:**

A total of 679 CBCT images from 97 patients were acquired for analysis. The mean setup errors for the whole group were 2.32 ± 1.21 mm, 3.71 ± 2.21 mm and 2.75 ± 1.56 mm in the LR, SI and AP directions, respectively. Patients’ BMI, CC and BV were moderately associated with setup errors, especially in the SI directions (R = 0.40, 0.43 and 0.22, respectively). Setup errors in the SI directions for patients with BMI > 23.8 kg/m^2^, CC > 89 cm and BV > 657 cm^3^ were 4.56 ± 2.59 mm, 4.77 ± 2.42 mm and 4.30 ± 2.43 mm, respectively, which were significantly greater than those of patients with BMI ≤ 23.8 kg/m^2^, CC ≤ 89 cm and BV ≤ 657 cm^3^ (*P* < 0.05). Correspondingly, the calculated PTV margins in patients with BMI > 23.8 kg/m^2^, CC > 89 cm and BV > 657 cm^3^ were 4.25/7.95/4.93 mm, 4.37/7.66/5.24 mm and 4.22/7.54/5.29 mm in the LR/SI/AP directions, respectively, compared with 3.64/4.64/5.09 mm, 3.31/4.50/4.82 mm and 3.29/5.74/4.73 mm for BMI ≤ 23.8 kg/m^2^, CC ≤ 89 cm and BV ≤ 657 cm^3^, respectively.

**Conclusions:**

The magnitude of geometric setup errors was moderately correlated with BMI, CC and BV. It was recommended to set patient-specific PTV margins according to patient characteristics in the absence of daily image-guided treatment setup.

## Background

Whole breast irradiation (WBI) after breast conserving surgery has become an important treatment for early-stage breast cancer patients and locally advanced breast cancer patients after effective preoperative systematic therapy [1–3]. Intensity-modulated radiation therapy (IMRT) is a widely used radiotherapy (RT) technique in WBI because of its greater dosimetric advantages and conformity compared with 2-dimensional and 3-dimensional (3D) conformal RT techniques [4]. The dose distributions of IMRT delivered to the target volume can be highly conformal with steep dose gradients. Thus it requires stringent treatment verification to ensure planning target volume (PTV) coverage is accurately maintained while the doses to organs at risk (OARs) are within the tolerance [5]. In breast RT, many setup error sources contribute to deviations from the planned dose distribution, such as setup inaccuracies of the whole patient and breast deformation [6]. For accurate dose delivery in RT, it is essential to correct target positioning with the help of image-guided radiation therapy (IGRT) techniques.

Kilovoltage cone-beam computed tomography (CBCT) is an IGRT technique that provides accurate 3D anatomical information for patient positioning for multiple disease sites. Additionally, it helps permit daily volumetric quality assurance of breast setups throughout the entire treatment delivery course [7]. It is generally used as a gold standard in stereotactic body radiation therapy and accelerated partial breast irradiation (APBI), which are delivered with a large-fraction dose or within only a few fractions [8, 9]. However, current CBCT techniques may not be optimal for WBI treatments in terms of radiation dose, acquisition time, and geometric clearance [10]. Thus, PTV, with appropriate margins from clinical target volume (CTV), would adequately account for geometric setup errors in the absence of daily IGRT. The Radiation Therapy Oncology Group (RTOG) recommended that the margin from CTV to PTV was a uniform 7 mm in the 1304 protocol [11, 12]. However, it is still controversial regarding whether the protocol-required uniform margin around the CTV adequately accounts for setup inaccuracies. The uncertainties need to be managed more precisely as the magnitude of setup inaccuracies was not uniform in each direction and varies with patient characteristics, or tumor cavity location [13–17]. Moreover, predictive factors for the magnitude of setup errors from these patient characteristics, including age, tumor location, and body mass index (BMI), chest circumference (CC) and breast volume (BV), may help to determine the adequate margins during treatment planning [16]. However, few previous studies have quantified daily setup uncertainties for the entire breast using CBCT data. The majority of studies were not based on population studies, and these predictive factors with setup inaccuracies have not been properly addressed [18, 19]. In the present study, we aimed to use CBCT imaging to determine the impacts of patient characteristics on the magnitude of geometric setup errors in a broader group of patients with WBI. Furthermore, a notable difference from previous work is that we analyzed patient groups according to correlative patient characteristics and obtained patient-specific PTV margins.

## Methods and materials

### Patients

A total of 97 patients with breast cancer (45 with left-sided and 52 with right-sided cancer) were consecutively enrolled in this study between January 2019 and December 2019. The study was approved by the Institutional Review Board of our institute. All patients had undergone breast-conserving surgery, followed by WBI.

### Treatment simulation and planning

For both simulation and treatment, all patients were immobilized in a Klarity Solo Align™ baseplate in the supine position with the head turned away from the treated side and arms stretched above the head. CT images were acquired from the upper neck to the upper abdomen with 5-mm-thick slices and imported to the treatment planning system. The simulation and treatment were performed with free breathing. Skin marks were applied to enable laser-based patient positioning before treatment. According to the RTOG 1304 protocol, the attending physicians delineated the target area and OARs for all patients. The target area included the breast CTV, which was limited anteriorly within 5 mm from the skin and posteriorly to the anterior surface of the pectoralis muscles, serratus anterior muscle/chestwall, boney thorax, and lung. Additionally, 7-mm margins around CTV were provided for PTV to compensate for the variability in treatment setup. OARs were also defined, such as the heart, lung, and contralateral whole breast.

In total, 5–7 beams were applied in IMRT techniques. Breast tangent fields with a 15–20-degree steeper gantry angle for medial beams were used. The prescription dose delivered to the whole breast was 50 Gy/25Fx and the tumor bed was boosted (10 Gy/5Fx) according to patients’ disease stage, age and tumor grade. The treatment goals were that 100% of the prescribed dose would cover 95% of PTV and the maximum dose would not exceed 105%.

### CBCT image acquisition and registration

During the whole breast treatment course, CBCT scans were immediately acquired after patients were positioned and were based on skin-marks for the first three treatment factions and weekly for the subsequent fractions using an Elekta Synergy X-ray volume imaging system (Elekta, Stockholm, Sweden). The scan protocol incorporated a 200° rotation at full gantry speed in a clockwise direction, which resulted in an acquisition time of 35 s. The kilovoltage exposure was set as 120 kV, 25 mA, and 40 ms, delivering an approximately dose of 3 mGy per scan.

In clinical practice at our institute, CBCT scans were routinely and automatically registered to the reference planning CT using the gray value algorithm, followed by a manual adjustment to obtain a better match on the breast, thoracic wall beneath the breast, and structures in the vicinity (a small volume of lung or heart) to obtain translational target errors in the LR, SI and AP directions. Setup errors were defined as the offset between CBCT and planning CT. The translational target errors were converted to a respective couch shift. All the rotational errors were disregarded due to limitations of couch movement.

### Patient characteristics for setup errors

Patient characteristics including patients’ age, tumor location, BMI, CC and BV were collected to analyze the correlation with setup errors. For continuous variables, the patients were divided into two groups above and below the median value, respectively. For tumor location, the patients were divided into left and right groups.

### Calculation of the PTV margin

Average displacements and standard deviation (SD) in three directions were calculated for each patient. The systematic error (Σ) was calculated from the SD of all average displacement for each patient, and the random error (σ) was the root mean square of all SD values for each patient. The CTV–PTV margins were calculated based on the calculation method described by van Herk [20]: 2.5Σ + 0.7σ.

### Statistical analysis

All statistical analyses were performed with IBM-SPSS statistics, version 19 (SPSS Inc, Chicago, IL). Linear regression analysis was used to separately assess the correlation between patient characteristics and setup errors in the three orthogonal directions. Correlations between setup errors and patient characteristics were analyzed with Pearson product-moment correlation coefficient (R). Furthermore, Setup errors between different groups were compared using t-test. *P* < 0.05 was considered to be statistically significant.

## Results

### Patient characteristics

In total, 97 patients were included in the study. The median (range) age, body weight, BMI were 47 (29–71) years, 61.0 (48.0–75.5) kg and 23.8 (17.4–29.6) kg/m^2^, respectively. No patients’ weight changed more than 2% during the treatment. The median (range) CC and BV were 89 (74–107) cm and 657 (264–1438) cm^3^, respectively. Patient characteristics are shown in Table 1. All patients received breast-conserving surgery, 81 patients received sentinel lymph node biopsy and 11 patients received axillary lymph nodes dissection. 50 Gy/25Fx irradiation was delivered to the whole breast with (n = 94) or without (n = 3) 10 Gy/5Fx boost to the tumor bed. No regional lymph node areas were irradiated.

**Table 1:** Patients characteristics and treatments

Characteristics	Median (range) or number of patients/n
Age (y)	47 (29–71)
Weight (kg)	61.0 (48.0–75.5)
BMI (kg/m^2^)	23.8 (17.4–29.6)
Chest circumference (cm)	89 (74–107)
Breast volume (cm^3^)	657 (264–1438)
Breast laterality	
Left-sided	45
Right-sided	52
Stage	
DCIS	5
I	53
II	39
Molecular subtypes	
Luminal A	39
Luminal B	24
TNBC	21
HER2 Overexpress	13
Surgery	
BCS	5
BCS + SLNB	81
BCS + ALND	11
Adjuvant chemotherapy	
Yes	66
No	31
Herceptin therapy	
Yes	21
No	76
Endocrine therapy	
Yes	64
No	33
Radiotherapy volume and dose	
Whole breast(50 Gy/25Fx)	3
Whole breast + tumor bed boost (60 Gy/30Fx)	94

*BMI* body mass index, *DCIS* ductal carcinoma in situ, *TNBC* triple negative breast cancer, *BCS* breast conserving surgery, *SLNB* sentinel lymph node biopsy, *ALND* axillary lymph nodes dissection

### Correlation between patient characteristics and geometric setup errors

**Fig. 1:**
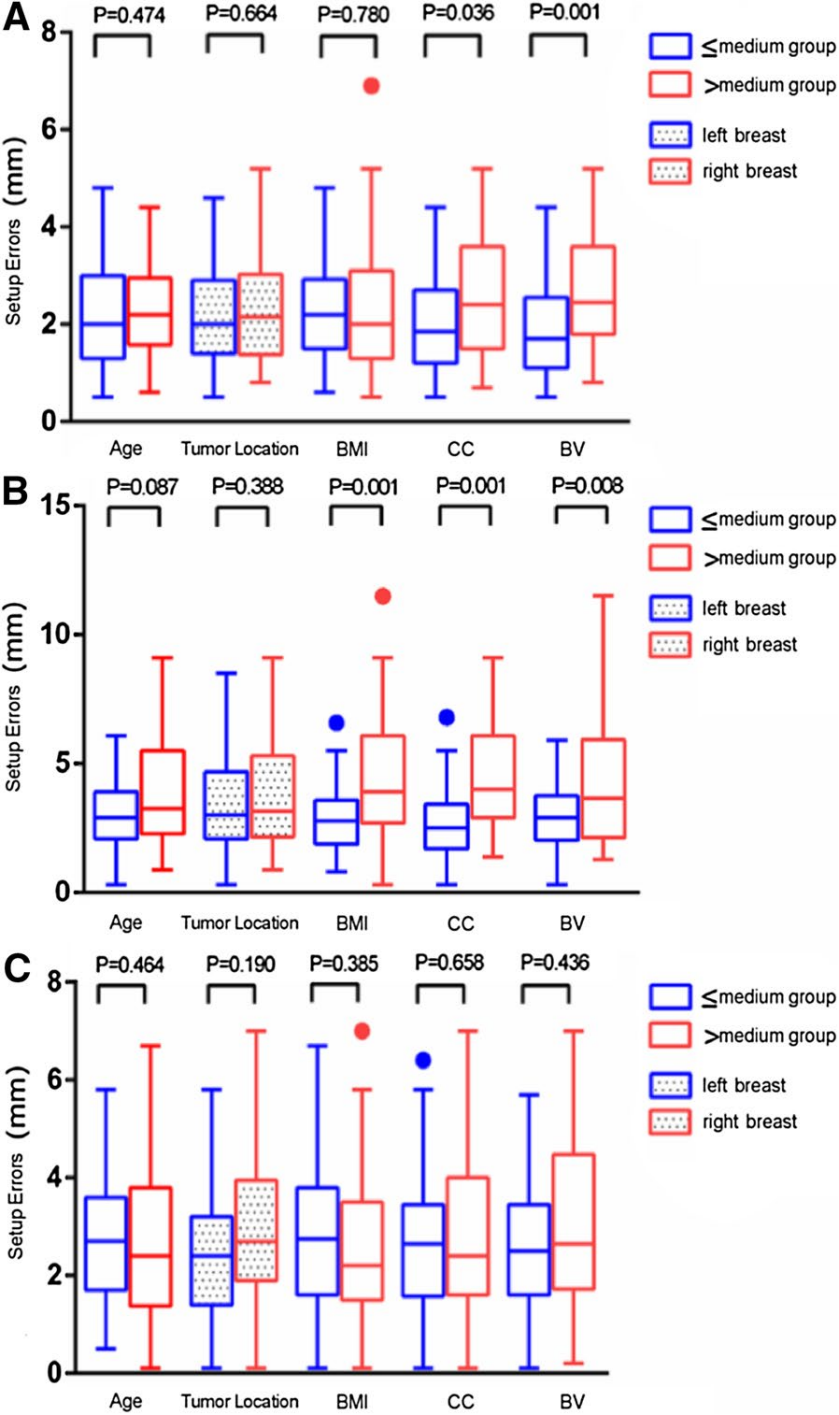
Setup errors between groups with different characteristics in LR (**A**), SI (**B**) and AP (**C**) directions

CBCT was taken in the first 3 days of treatment, and then once weekly throughout the whole breast treatment course. A total of 679 CBCT images from 97 patients were acquired for analysis. For the whole group, the mean geometric setup errors were 2.32 ± 1.21 mm, 3.71 ± 2.21 mm and 2.75 ± 1.56 mm in the LR, SI and AP directions, respectively. The correlation between patient characteristics and translational setup errors is summarized in Table 2. Patients’ age (Table 2) and breast laterality (Fig. 1) were found to have no influence on the setup errors in all directions. Conversely, the BMI was moderately associated with the setup errors of SI directions (R = 0.40). In addition, CC and BV were correlated with the magnitude of setup errors in the SI directions (R = 0.43 and 0.22, respectively). The magnitude of setup errors in the LR directions was also moderately correlated with BV (R = 0.42). No significant correlations were found between setup errors in the AP directions and patients’ age, BMI, CC or CV.

**Table 2:** Correlation coefficients between patient characteristics and translational setup errors

Characteristics	Median (range)	Correlation coefficients		
		LR	SI	AP
Age (years)	47 (29–71)	0.03	0.14	− 0.01
BMI (kg/m^2^)	23.8 (17.4–29.6)	− 0.07	0.40*	0.01
CC (cm)	89 (74–107)	0.19	0.43*	0.08
BV (cm^3^)	657 (264–1438)	0.42*	0.22*	0.06

*LR* left–right direction, *SI* superior–inferior direction, *AP* anterior–posterior direction, *BMI* body mass index, *CC* chest circumference, *BV* breast volume

*P < 0.05

### Comparison of setup errors between groups with different patient characteristics

To better demonstrate the association between the magnitudes of setup errors and patient characteristics, all patients were divided into two groups on the basis of the median values. Setup errors between the groups with different characteristics are shown in Fig. 1. Higher BMI, broader CC and larger BV patients have significantly greater setup uncertainties, especially in the SI directions. For patients with BMI > 23.8 kg/m^2^, CC > 89 cm and BV > 657 cm^3^, setup errors in the SI directions were 4.56 ± 2.59 mm, 4.77 ± 2.42 mm and 4.30 ± 2.43 mm, compared with 2.91 ± 1.33 mm, 2.71 ± 1.34 mm and 3.13 ± 1.75 mm for patients with BMI ≤ 23.8 kg/m^2^, CC ≤ 89 cm and BV ≤ 657 cm^3^, respectively (*P* < 0.05) (Fig. 1B). Similar results were observed in the LR directions (Fig. 1A). There was no difference in setup errors for the AP directions between different groups (Fig. 1C). Consistent with the results of the correlation analysis, no difference in setup errors was observed between patients with age > 46 years and those with age ≤ 46 years in all directions, which was also the case for breast laterality (left-sided vs. right-sided) (Fig. 1).

### Patient-specific PTV margins according to patient characteristics

According to the results, the magnitudes of setup inaccuracies were not uniform but varied with directions and patient characteristics. For accurate management of uncertainties, we calculated the margins for all patients. PTV margins according to patient characteristics are shown in Table 3. Consistent with the results of setup errors in different groups, patients with a higher BMI, broader CC and larger BV who have greater setup uncertainties need larger margins to ensure greater coverage of the targets. The margins in the LR/SI/AP directions were 4.25/7.95/4.93 mm for patients with BMI ≥ 23.8 kg/m^2^ and 3.64/4.64/5.09 mm for patients with BMI < 23.8 kg/m^2^, respectively. Similarly, for patients with CC > 89 cm and BV > 657 cm^3^, the margins were 4.37/7.66/5.24 mm and 4.22/7.54/5.29 mm, respectively, which were much larger than those of patients with CC ≤ 89 cm and BV ≤ 657 cm^3^ with margins of 3.31/4.50/4.82 mm and 3.29/5.74/4.73 mm, respectively.

**Table 3:** Calculated CTV–PTV margins in 3-dimensions of different characteristics

Groups	Margins (mm)		
	LR	SI	AP
Age > 46 years	3.98	7.13	4.99
Age ≤ 46 years	3.93	6.52	5.03
BMI > 23.8 kg/m^2^	4.25	7.95	4.93
BMI ≤ 23.8 kg/m^2^	3.64	4.64	5.09
CC > 89 cm	4.37	7.66	5.24
CC ≤ 89 cm	3.31	4.50	4.82
BV > 657 cm^3^	4.22	7.54	5.29
BV ≤ 657 cm^3^	3.29	5.74	4.73

*LR* left–right direction, *SI* superior–inferior direction, *AP* anterior–posterior direction, *BMI* body mass index, *CC* chest circumference, *BV* breast volume

## Discussion

As a feature of IMRT, accurate patient positioning is of great importance for precise breast RT. Patient position accuracy is affected by various factors. In our study, we evaluated the correlation between set up errors and patient characteristics, including patients’ age, breast laterality, BMI, CC and BV. We found that patients’ age and breast laterality had no correlation with setup errors in all three directions. Conversely, BMI and CC and BV were moderately associated with SI direction setup errors. Beyond that finding, a larger BV was also correlated with LR direction setup errors. Based on the impact of patient characteristics on setup errors, we calculated patient-specific PTV margins according to patient characteristics to adequately compensate setup uncertainties. To our knowledge, the present study is one of a few studies based on a large Asian population that evaluates the correlation between patient characteristics and geometric setup errors, meanwhile offering patient-specific PTV margins according to correlative characteristics in WBI with CBCT images.

In the present study, age was not associated with setup errors in all directions. This is consistent with the results of Mulliez et al. who reported that setup errors and PTV margins were not influenced by age, neither in the prone nor supine position [14]. In addition, in Hirata et al.’s study [15], age was also found to have no correlation with respiratory-induced motion or baseline drift in APBI. Thus, age might have little influence on setup errors, although the elderly patients are considered to have lower skin elasticity and skin turgor [21], which might have induced more uncertainties when patients are positioned according to the skin markers. Beyond that, we also compared setup errors between treatments of left and right-sided tumors and found that setup errors between left- and right-sided breast cancer patients were similar (Fig. 1). One of the factors that may influence left-sided breast cancer patients’ setup uncertainty is heartbeat. Topolnjak et al. quantified the geometrical uncertainties for the heart and found considerable cardiac position variability during RT in left-sided breast cancer patients [22]. Thus, heart position uncertainty can be mitigated by using OAR volume margins during treatment planning. In our study, we did not evaluate the position variability of the heart but analyzed the influence of heartbeats on setup errors by comparing left- and right-sided breast cancer patients. Uncertainties were similar, indicating that heartbeats had little influence on setup errors during WBI.

The effect of BMI on setup errors during RT has actually been reported in many cancers [23, 24]. In our study, BMI, CC and BV were significant correlated with SI direction setup errors with the correlation coefficients of 0.40, 0.43 and 0.22, respectively. The finding that BMI was correlated with setup errors was partially similar to that of the studies by Mulliez et al. and Lee et al. [14, 16]. Mulliez et al. reported that BMI was the only determinant factor to have a significant influence on the SI margins. Additionally, with the increase in cup size, increased LR margins were observed [14]. Lee et al. observed that the interfraction setup errors in reverse semidecubitus breast RT were correlated with high body weight and BMI in the AP direction, but not the SI or LR directions. The mean setup errors were − 1.1 ± 3.0 and − 4.0 ± 2.9 mm in the AP direction for patients with BMI ≤ 25.9 and > 25.9, respectively (*P* = 0.01) [16]. They found that the larger interfraction drifts in the AP direction for high body weight and BMI patients were attributed to the compression of back subcutaneous fat or muscle relaxation. In our study, BMI, CC and BV significantly influenced the SI direction but not AP direction errors. The reasons that contributed to the different results included ethnic differences, different patient positions and immobilization devices. Patients in our study were positioned with both upper limbs outstretched and raised overhead. High BMI, broad CC and large BV patients had a thicker skin and breast fat layer, which could induce larger variability in the SI direction, but not the LR or AP direction in that position. Further more, 5-mm-thick slices planning CT images were applied in our study, which might reduce the resolution and influence the matches between planning CT and CBCT images. This is also an important factor that increased the SI setup errors.

Patients with larger breasts are likely to present larger errors, because soft tissue breasts are highly deformable and susceptible to movement. It is also noticeable that CC and BV are intrinsically correlated, because a broader CC is usually observed in patients with larger BV. Since the breast is not a rigid structure, the deformation or movement of fat or muscle relaxation may be more significant in larger-breasted patients, leading to larger setup errors [13]. Breast deformation during the course of RT is also an important factor that influences the set up errors. That maximum breast surface expansion (MBSE) could reach 15 mm, and MBSE ≥ 5 mm were observed in 17% fractions during the course of RT [13]. Alteration in breast shape causes local misalignments which needs deformable registration. However, it is quite difficult to quantify breast deformation from the available CBCTs in the absence of deformable registration in our current version of X-ray volume imaging system. In addition, setup errors were corrected using couch shifts through the rigid registration in our study. Thus, the breast deformation was not focused in the present study.

Apart from the factors discussed above, respiratory motion is also an important aspect that influences the setup errors. The medial field border of WBI for the normal inhalation and normal exhalation scans were reported to move average of 6 mm anteriorly and 3 mm posteriorly compared with the free-breathing position, respectively [25]. Ono et al. reported that the AP motion was 1.36 ± 0.94 (0.14–5.28) mm in left-sided breast cancer patients treated with WBI using deep inspiration breath-hold [26]. In our study, no method of controlling for respiration was applied and the set up errors in AP direction was 2.75 ± 1.56 cm in free breathing, which was much lager than Yuka Ono et al. reported [26]. So the PTV margins might be further reduced if the breath- hold techniques are used in conjunction with image guidance. Howsoever, the effects of respiratory motion during fractionated radiation therapy still need to be further investigated.

Based on setup error results, PTV margins according to patient characteristics were calculated using the method described in the van Herk formula [20]. According to the results in Table 3, approximately 7–8 mm margins in the SI directions were needed for patients with BMI > 23.8 kg/m^2^, CC > 89 cm or BV > 657 cm^3^, which were close to the 7-mm margins recommended by the RTOG protocol. However, patients with lower BMI (≤ 23.8 kg/m^2^), smaller CC (≤ 89 cm) and BV (≤ 657cm^3^) only required less than 6-mm margins in the SI directions. Additionally, approximately 4–5-mm margins in the LR and AP directions might be enough to ensure the adequate PTV coverage for all patients. Compared with the calculated margins in WBI with CBCT of previous studies, the present results were similar with Donovan et al.’s [18] and van Mourik et al.’s [6] studies, which reported calculated margins of 5.9/5.0/5.2 mm and 5.9/5.5/5.9 mm in the LR/SI/AP directions, respectively. However, in Kirby et al.’s [27], Veldeman et al.’s [28] and Mulliez et al.’s [14] studies, the estimated margins were approximately 10 mm in each direction. Various reasons—such as different races, sample sizes, verification protocols, positioning and immobilization devices—may contribute to these differences in calculated margins. In addition, the results were slightly different from the RTOG-recommended symmetrical 7-mm PTV margins. The most important reason could be due to ethnic differences, as the BMI of Asian populations is much lower than those of American and European populations [29]. Additionally, the breast sizes between Asian and American or European populations varied greatly. The breast sizes in mammography were reported to be 168.4 cm^2^ in African-American women and 121.7 cm^2^ in white women [30]. However, in Asian women, the average breast size was 102.3 cm^2^ in mammography [31]. Due to the great difference in BMI and breast size between Asian and American or European women, the RTOG recommended PTV margins might not be completely appropriate for Asian populations. According to our results, compared with American or European populations, smaller PTV margins are probably more suitable for Asian populations.

In recent years, surface guided radiation therapy (SGRT) using optical surface scanning has become an alternative tool in RT due to the ability to provide 6-dimensional motion monitoring of the patient surface in real-time using a non-invasive approach, without any additional radiation exposure [32]. The most promising application is gated RT delivery of superficial tumors (e.g. breast cancer). Only surface data are available for SGRT. It leads the fact that information concerning geometrical uncertainties in surrounding OARs is lacking and differences between anatomical changes or setup errors are impossible to distinguish. Routine CBCT is still considered the gold standard in current clinical practice. Surface imaging for setup verification can be an important addition to CBCT, which may reduce the frequency of CBCT verification and facilitate patient-specific QA.

There are some limitations in this study. Firstly, we only conducted the pretreatment CBCT, and the primary focus was the appraisal of interfraction setup errors. No attempts have been made to explore the domain of intrafraction setup errors in our analysis because posttreatment CBCT was not acquired. In other published work, intrafraction fluctuations and their effect on setup errors were generally small compared with interfraction motion [33]. The frequency of performing CBCT throughout RT treatment depends on the local practice of the individual institution and the complexity of the patient’s treatment. Hansen et al. reported their experience using daily CBCT and found mean setup deviations of 3.6, 4.1, and 1.0 mm in the RL, SI, and AP axis, respectively. Use of daily CBCT allows reduced PTV expansions owing to less uncertainty in random geometric errors and may confer an obvious benefit particularly for larger patients [34]. However, it adds a considerable amount of radiation dose for the patient, thereby increasing the risks of inducing secondary cancers [10, 35]. In this study, CBCT scans were acquired for the first three factions to ensure that the treatment was safe and delivered as planned. Then, CBCT was typically used as verification on a weekly basis. A balance can be achieved between the needs of the imaging dose and resource requirements. Secondly, the information from rotational errors was not integrated. CBCT data provided increased sensitivity for rotational setup errors. The local practice is to correct for large rotations (> 3°) by repeating a skin-mark setup. Small rotations have been demonstrated to result in translational inaccuracies [36]; however, this was not explicitly corrected due to the absence of the 6-degree treatment couch. If they can be accurately corrected, we speculate the actual margins may be less.

Despite these limitations, our study provides a strong indication of interpatient variability and an accurate representation of the treatment anatomy. Generally, we found that the 4–5-mm PTV margins for the AP and LR directions were enough to ensure the target whole breast was fully irradiated for all patients. The 7–8-mm PTV margins for the SI directions were necessary for patients with BMI > 23.8 kg/m^2^, CC > 89 cm or BV > 657cm^3^. These margins would adequately cover the PTV and account for setup errors in the absence of daily IGRT.

## Conclusion

This study evaluated geometric setup errors with CBCT in WBI with free breathing. The magnitude of geometric setup errors was moderately correlated with BMI, CC and BV. It was recommended to set patient-specific PTV margins according to patient characteristics in the absence of daily image-guided treatment setup. For larger patients, daily imaging may be particularly important to minimize the geometric uncertainty. A standardized, accurate, safe, timely, and cost-effective IGRT protocol is essential for further investigation.

## Data Availability

The datasets used and/or analysed during the current study are available from the corresponding author on reasonable request.

## Abbreviations

CBCT: Cone-beam computed tomography
WBI: Whole breast irradiation
LR: Left–right
SI: Superior–inferior
AP: Anterior–posterior
BMI: Body mass index
CC: Chest circumference
BV: Breast volume
PTV: Planning target volume
IMRT: Intensity-modulated radiation therapy
RT: Radiotherapy
OARs: Organs at risk
IGRT: Image-guided radiation therapy
APBI: Accelerated partial breast irradiation
CTV: Clinical target volume
RTOG: Radiation Therapy Oncology Group
SGRT: Surface guided radiation therapy

## References

[1] Veronesi U, Luini A, Del Vecchio M, Greco M, Galimberti V, Merson M (1993). Radiotherapy after breast-preserving surgery in women with localized cancer of the breast. N Engl J Med.

[2] Fisher B, Anderson S, Bryant J, Margolese RG, Deutsch M, Fisher ER (2002). Twenty-year follow-up of a randomized trial comparing total mastectomy, lumpectomy, and lumpectomy plus irradiation for the treatment of invasive breast cancer. N Engl J Med.

[3] Kaidar-Person O, Kuten A, Belkacemi Y, Arome O (2014). Primary systemic therapy and whole breast irradiation for locally advanced breast cancer: a systematic review. Crit Rev Oncol Hematol.

[4] Jain P, Marchant T, Green M, Watkins G, Davies J, McCarthy C (2009). Inter-fraction motion and dosimetric consequences during breast intensity-modulated radiotherapy (IMRT). Radiother Oncol.

[5] Beckham WA, Popescu CC, Patenaude VV, Wai ES, Olivotto IA (2007). Is multibeam IMRT better than standard treatment for patients with left-sided breast cancer?. Int J Radiat Oncol Biol Phys.

[6] van Mourik A, van Kranen S, den Hollander S, Sonke JJ, van Herk M, van Vliet-Vroegindeweij C (2011). Effects of setup errors and shape changes on breast radiotherapy. Int J Radiat Oncol Biol Phys.

[7] White EA, Cho J, Vallis KA, Sharpe MB, Lee G, Blackburn H (2007). Cone beam computed tomography guidance for setup of patients receiving accelerated partial breast irradiation. Int J Radiat Oncol Biol Phys.

[8] Fatunase T, Wang Z, Yoo S, Hubbs JL, Prosnitz RG, Yin FF (2008). Assessment of the residual error in soft tissue setup in patients undergoing partial breast irradiation: results of a prospective study using cone-beam computed tomography. Int J Radiat Oncol Biol Phys.

[9] Du S, Lockamy V, Zhou L, Xue C, LeBlanc J, Glenn S (2016). Stereotactic body radiation therapy delivery in a genetically engineered mouse model of lung cancer. Int J Radiat Oncol Biol Phys.

[10] Kan MW, Leung LH, Wong W, Lam N (2008). Radiation dose from cone beam computed tomography for image-guided radiation therapy. Int J Radiat Oncol Biol Phys.

[11] Goddu SM, Yaddanapudi S, Pechenaya OL, Chaudhari SR, Klein EE, Khullar D (2009). Dosimetric consequences of uncorrected setup errors in helical tomotherapy treatments of breast-cancer patients. Radiother Oncol.

[12] NCT01872975. A randomized phase III clinical trial evaluating post-mastectomy chestwall and regional nodal XRT and post-lumpectomy regional nodal XRT in patients with positive axillary nodes before neoadjuvant chemotherapy who convert to pathologically negative axillary nodes after neoadjuvant chemotherapy. Available from https://clinicaltrials.gov/ct2/show/NCT01872975. Accessed 31 Aug 2020.

[13] Seppala J, Vuolukka K, Viren T, Heikkila J, Honkanen JTJ, Pandey A (2019). Breast deformation during the course of radiotherapy: the need for an additional outer margin. Phys Med.

[14] Mulliez T, Gulyban A, Vercauteren T, van Greveling A, Speleers B, De Neve W (2016). Setup accuracy for prone and supine whole breast irradiation. Strahlenther Onkol.

[15] Hirata K, Yoshimura M, Mukumoto N, Nakamura M, Inoue M, Sasaki M (2017). Three-dimensional intrafractional internal target motions in accelerated partial breast irradiation using three-dimensional conformal external beam radiotherapy. Radiother Oncol.

[16] Lee J, Liu SH, Lin JB, Wu MH, Wu CJ, Tai HC (2018). Image-guided study of inter-fraction and intra-fraction set-up variability and margins in reverse semi-decubitus breast radiotherapy. Radiat Oncol.

[17] Zhen X, Zhao B, Wang Z, Timmerman R, Spangler A, Kim N (2017). Comprehensive target geometric errors and margin assessment in stereotactic partial breast irradiation. Radiat Oncol.

[18] Donovan EM, Castellano I, Eagle S, Harris E (2012). Clinical implementation of kilovoltage cone beam CT for the verification of sequential and integrated photon boost treatments for breast cancer patients. Br J Radiol.

[19] Topolnjak R, de Ruiter P, Remeijer P, van Vliet-Vroegindeweij C, Rasch C, Sonke JJ (2011). Image-guided radiotherapy for breast cancer patients: surgical clips as surrogate for breast excision cavity. Int J Radiat Oncol Biol Phys.

[20] van Herk M, Remeijer P, Rasch C, Lebesque JV (2000). The probability of correct target dosage: dose-population histograms for deriving treatment margins in radiotherapy. Int J Radiat Oncol Biol Phys.

[21] Gerhardt LC, Lenz A, Spencer ND, Munzer T, Derler S (2009). Skin-textile friction and skin elasticity in young and aged persons. Skin Res Technol.

[22] Topolnjak R, Borst GR, Nijkamp J, Sonke JJ (2012). Image-guided radiotherapy for left-sided breast cancer patients: geometrical uncertainty of the heart. Int J Radiat Oncol Biol Phys.

[23] Lin LL, Hertan L, Rengan R, Teo BK (2012). Effect of body mass index on magnitude of setup errors in patients treated with adjuvant radiotherapy for endometrial cancer with daily image guidance. Int J Radiat Oncol Biol Phys.

[24] Wong JR, Gao Z, Merrick S, Wilson P, Uematsu M, Woo K (2009). Potential for higher treatment failure in obese patients: correlation of elevated body mass index and increased daily prostate deviations from the radiation beam isocenters in an analysis of 1,465 computed tomographic images. Int J Radiat Oncol Biol Phys.

[25] Frazier RC, Vicini FA, Sharpe MB, Yan D, Fayad J, Baglan KL (2004). Impact of breathing motion on whole breast radiotherapy: a dosimetric analysis using active breathing control. Int J Radiat Oncol Biol Phys.

[26] Ono Y, Yoshimura M, Ono T, Fujimoto T, Miyabe Y, Matsuo Y (2021). Appropriate margin for planning target volume for breast radiotherapy during deep inspiration breath-hold by variance component analysis. Radiat Oncol.

[27] Kirby AM, Evans PM, Donovan EM, Convery HM, Haviland JS, Yarnold JR (2010). Prone versus supine positioning for whole and partial-breast radiotherapy: a comparison of non-target tissue dosimetry. Radiother Oncol.

[28] Veldeman L, De Gersem W, Speleers B, Truyens B, Van Greveling A, Van den Broecke R (2012). Alternated prone and supine whole-breast irradiation using IMRT: setup precision, respiratory movement and treatment time. Int J Radiat Oncol Biol Phys.

[29] NCD Risk Factor Collaboration (NCD-RisC) (2016). Trends in adult body-mass index in 200 countries from 1975 to 2014: a pooled analysis of 1698 population-based measurement studies with 19.2 million participants. Lancet.

[30] Stuedal A, Ma H, Bernstein L, Pike MC, Ursin G (2008). Does breast size modify the association between mammographic density and breast cancer risk?. Cancer Epidemiol Biomark Prev.

[31] Lim LY, Ho PJ, Liu J, Chay WY, Tan MH, Hartman M (2018). Determinants of breast size in Asian women. Sci Rep.

[32] Hattel SH, Andersen PA, Wahlstedt IH, Damkjaer S, Saini A, Thomsen JB (2019). Evaluation of setup and intrafraction motion for surface guided whole-breast cancer radiotherapy. J Appl Clin Med Phys.

[33] Smith RP, Bloch P, Harris EE, McDonough J, Sarkar A, Kassaee A (2005). Analysis of interfraction and intrafraction variation during tangential breast irradiation with an electronic portal imaging device. Int J Radiat Oncol Biol Phys.

[34] Hansen EK, Larson DA, Aubin M, Chen J, Descovich M, Gillis AM (2006). Image-guided radiotherapy using megavoltage cone-beam computed tomography for treatment of paraspinous tumors in the presence of orthopedic hardware. Int J Radiat Oncol Biol Phys.

[35] Cheng HC, Wu VW, Liu ES, Kwong DL (2011). Evaluation of radiation dose and image quality for the varian cone beam computed tomography system. Int J Radiat Oncol Biol Phys.

[36] Ezzell LC, Hansen EK, Quivey JM, Xia P (2007). Detection of treatment setup errors between two CT scans for patients with head and neck cancer. Med Phys.

